# Cognitive rehabilitation in people with autism spectrum disorder: a systematic review of emerging virtual reality-based approaches

**DOI:** 10.1186/s12984-022-01069-5

**Published:** 2022-08-18

**Authors:** Leila Shahmoradi, Sorayya Rezayi

**Affiliations:** Health Information Management and Medical Informatics Department, School of Allied Medical Sciences, Tehran University of Medical Sciences, Tehran, Iran

**Keywords:** Virtual reality, Autism spectrum disorder, Cognitive rehabilitation, Virtual reality-based cognitive rehabilitation

## Abstract

**Introduction:**

Emerging virtual technologies and cognitive rehabilitation methods are two new treatment approaches that can be used to strengthen cognitive functions in Autism Spectrum Disorder (ASD). The main aim of this study was to examine the effect of using virtual reality-based approaches on cognitive disorders of children and adults with ASD.

**Methods:**

This systematic review was conducted on scientific papers to determine the effects of virtual reality-based technologies on the cognitive functions of children and adults with ASD. We identified 688 studies related to this topic and filtered them down to 17 articles, and then extracted the effects of interventions on cognitive outcomes.

**Results:**

A total of 17 studies met the inclusion criteria, in which 226 persons with ASD had taken place. The sample size in the selected studies ranged from 1 to 56 participants (Median: 8, Q1: 3.5, Q3: 15.5). Four of the studies were case–control studies, ten were pre-test/post-test studies, and three were Randomized Control Trials (RCTs). Results of 16 studies showed significant progress in various cognitive indexes, such as task learning, attention, executive functioning, and daily skills in people with ASD. In most studies, virtual technologies had beneficial effects on reducing cognitive problems, but existing limitations could reduce their effectiveness. These limitations included the cost of virtual reality devices, inappropriate size of software, the weight of devices, potential addiction, intolerance of wearing glasses or headsets by people with autism (especially in children), and the possibility of eye injury.

**Conclusion:**

Applying appropriate virtual-based approaches could improve cognitive indexes in people with ASD. However, further studies are needed to investigate the real effects of these technologies in the long run.

## Introduction

Autism spectrum disorder (ASD) is a complex neurobehavioral disorder that involves impaired social interaction, verbal underdevelopment, problems with communication skills, and challenging and repetitive behaviors; ASD has a wide range of symptoms [1]. About 1 in 68 children are diagnosed with autism, and boys are more likely to have ASD than girls [2]. ASD is characterized by symptoms such as excessive activity, the problem with attention, decreased learning in school, and aggressive behaviors [3]. People with ASD have different cognitive and intelligence profiles than ordinary people [3, 4]. In addition to biological factors, environmental factors such as poverty, poor housing, low socioeconomic status, large families, incompatibility, conflicts between parents, and aggression in the family are some of the causes of ASD [5]. Several studies have shown that most children and adults with ASD have delays in their cognitive skills [6, 7]. Increasing awareness about cognitive phenotype will help to understand the better relationship between genes, brain, and behavior and provide more information about treatment methods [8]. Active memory is a crucial cognitive function in rehabilitating and evaluating individuals and children with special needs. Active memory is the cognitive executive/functional ability used for academic, behavioral, and social functions [9]. Meanwhile, active memory helps to store and process information. Many of the critical features and behavioral problems of autistic children and adults result from executive dysfunction. Executive function is a general term for mental abilities such as programming, working memory, impulse control, inhibition, transmission planning, and the ability to initiate and execute tasks [10]. This skill usually plays a vital role in one’s emotional, social, cognitive, and behavioral development. Therefore, if such disorders are evaluated and treated from childhood, many behavioral problems can be prevented in adulthood. Most families prefer to use cognitive rehabilitation services to solve their children's problems with executive functioning, attention, and memory, and also improve their learning and daily skills [11, 12]. Thus, it can be acknowledged that attention, memory, executive functioning and learning are the cognitive defects of children and adults with ASD, which can be enhanced by cognitive rehabilitation techniques [13].

Given the challenges that exist in improving the health status of children and adults, paying attention to emerging approaches to improve cognitive abilities seems to be a way forward. Cognitive rehabilitation includes a wide range of treatment methods that can be performed by different rehabilitation specialists [14]. Cognitive rehabilitation helps to restore normal functioning and compensate for cognitive deficits in people with brain damage or people with cognitive impairment [15].

Virtual technology refers to the technology that intends to imitate a physical world. This imitation is developed through the simulated or digital world by constructing a sensory feeling. Accordingly, this technology can create a sense of reality in people. There are three primary categories of virtual reality simulations, which include non-immersive, semi-immersive, and fully-immersive simulations [16]. All types of virtual technology are beneficial for sciences such as telemedicine, robot development, and computer-based rehabilitation [10]. Therefore, it would be safe to say that virtual reality technologies and cognitive rehabilitation are two new treatment approaches that promote the functions of patients in specific areas such as attention, memory, component function, and perceptual abilities. They do this by sensory involvement and increased visual and auditory feedback [17]. This technology has the potential to create scenarios in the field of cognitive rehabilitation that facilitate brain reconstruction [16]. According to our knowledge, no systematic review has been conducted to investigate the effects of virtual reality-based approaches on the cognitive outcomes of people with ASD.

### Objectives

The main aim of this study was to examine the effect of virtual reality-based technologies (non-immersive, semi-immersive, and fully-immersive simulation) on the cognitive disorders of people with ASD (children and adults). The specific aims of this review included:

A) Providing an overview of published papers and their critical characteristics,

B) Summarizing and excavating the selected citations,

C) Investigating the effects of virtual reality-based technologies on improving the cognitive functions of children and adults with ASD.

## Research methodology

This systematic review was conducted based on the Preferred Reporting Items for Systematic Reviews and Meta-Analyzes (PRISMA) method [18].

### Design

In this systematic review, a comprehensive and systematic search was performed in scientific papers published until April 09, 2021. A search with no time limitation was carried out in four scientific databases, including Medline (through PubMed), ISI Web of Science, Scopus, and IEEE Xplore. These databases were selected because of their qualitative and health research coverage. A set of keywords such as Emtree and Mesh related to virtual reality, cognition, cognitive rehabilitation and autism were used in the search strategy. The detail of search strategy for each database is presented in Table 1.

**Table 1:** Search strategy for each database

Database	Search strategy
PubMed	(“Virtual reality “[Mesh] OR “virtual immersive technology” OR “serious game” OR “virtual training” OR “virtual environment” OR “Virtual Game” OR “Virtual based game” OR “3-D game” OR “virtual train” OR "Virtual Reality Exposure Therapy"[Mesh] OR “Virtual Reality Immersion Therapy” OR “Virtual Reality Therapy”) AND ("Autistic Disorder"[Mesh] OR "Autism Spectrum Disorder"[Mesh] OR “Autism” OR “ Autistic child” OR “Autistic children” OR “Autistic disorder” OR “Autistic spectrum disorder” OR “Child development disorders” OR “Classical autism” OR “Early infantile autism” OR “Infantile autism” OR " Kanner syndrome" OR "Pervasive developmental disorder" OR "Typical autism" OR "Kanners Syndrome" OR "Kanner's Syndrome") AND ("Cognition" OR "Cognition Therapy" OR "Cognitive Dysfunction" OR "Cognitive Decline" OR "Cognitive Impairment" OR "cognitive task" OR "cognitive thinking" OR "cognitive rehabilitation" OR "Cognitive function" OR “attention” OR “executive function” OR “confusion” OR “imagination” OR “learning” OR “memory” OR “orientation” OR “thinking” OR “numerical cognition” OR “fantasy” OR “intuition” OR “perception” OR “cognitive reserve”) Results = 109
Web of Science	TS = (“Virtual reality” OR “virtual immersive technology” OR “serious game” OR “virtual training” OR “virtual environment” OR “Virtual Game” OR “Virtual based game” OR “virtual train” OR "Virtual Reality Exposure Therapy" OR “Virtual Reality Immersion Therapy” OR “Virtual Reality Therapy”) AND TS = ("Autistic Disorder" OR "Autism Spectrum Disorder" OR “Autism” OR “Autistic child” OR “Autistic children” OR “Autistic disorder” OR “Autistic spectrum disorder” OR “Child development disorders” OR “Classical autism” OR “Early infantile autism” OR “Infantile autism” OR " Kanner syndrome" OR "Pervasive developmental disorder" OR "Typical autism" OR "Kanners Syndrome") AND TS = ("Cognition" OR "Cognition Therapy" OR "Cognitive Dysfunction" OR "Cognitive Decline" OR "Cognitive Impairment" OR "cognitive task" OR "cognitive thinking" OR "cognitive rehabilitation" OR "Cognitive function" OR “attention” OR “executive function” OR “confusion” OR “imagination” OR “learning” OR “memory” OR “orientation” OR “thinking” OR “numerical cognition” OR “fantasy” OR “intuition” OR “perception” OR “cognitive reserve”) Results: 232
Scopus	(“Virtual reality” OR “virtual immersive technology” OR “serious game” OR “virtual training” OR “virtual environment” OR “Virtual Game” OR “Virtual based game” OR “virtual train” OR "Virtual Reality Exposure Therapy" OR “Virtual Reality Immersion Therapy” OR “Virtual Reality Therapy”) AND ("Autistic Disorder" OR "Autism Spectrum Disorder" OR “Autism” OR “Autistic child” OR “Autistic children” OR “Autistic disorder” OR “Autistic spectrum disorder” OR “Child development disorders” OR “Classical autism” OR “Early infantile autism” OR “Infantile autism” OR "Kanner syndrome" OR "Pervasive developmental disorder" OR "Typical autism" OR "Kanners Syndrome") AND ("Cognition" OR "Cognition Therapy" OR "Cognitive Dysfunction" OR "Cognitive Decline" OR "Cognitive Impairment" OR "cognitive task" OR "cognitive thinking" OR "cognitive rehabilitation" OR "Cognitive function" OR “attention” OR “executive function” OR “confusion” OR “imagination” OR “learning” OR “memory” OR “orientation” OR “thinking” OR “numerical cognition” OR “fantasy” OR “intuition” OR “perception” OR “cognitive reserve”) Results = 593
IEEE Xplore library	((“Virtual reality” OR “Virtual Game” OR “Virtual Reality Immersion Therapy” OR “Virtual Reality Therapy”) AND ("Autistic Disorder" OR "Autism Spectrum Disorder" OR “Autism”) AND ("Cognition" OR "Cognition Therapy" OR "Cognitive Dysfunction" OR "Cognitive Decline" OR "Cognitive Impairment")) Results = 20

### Inclusion and exclusion criteria

**Fig. 1:**
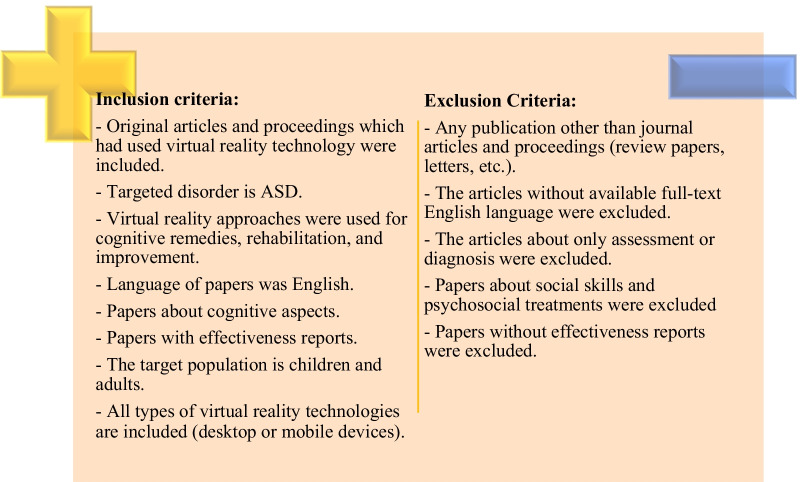
Exclusion and inclusion criteria used to select eligible articles

The selected academic papers were screened based on exclusion and inclusion criteria that are displayed in Fig. 1.

### Literature refinement

**Fig. 2:**
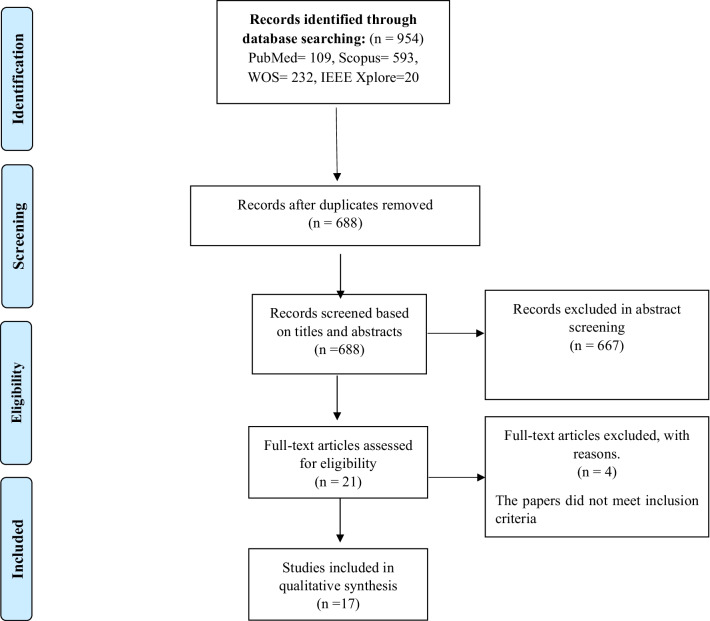
Flow diagram of the literature search and selection of articles

The scientific search resulted in the extraction of 688 papers after removal of duplicates. All abstracts and titles were evaluated based on the research questions and objectives to select relevant articles. Title and abstract screening led to the exclusion of 667 articles. In the first examination, 21 articles seemed relevant, and their full text was examined and reviewed. After examining the full text of these articles and applying the inclusion and exclusion criteria, 17 articles were included in this systematic review. Critical items in each article were entered into a spreadsheet in Excel. Two authors (SR and LS) independently extracted the study characteristics for each paper. This information was re-examined again by LS to reach an agreement. Screening and selecting procedures are presented in Fig. 2, based on the PRISMA method.

### Data analysis and synthesis

In this study, articles that investigated the effects of using virtual reality-based approaches on cognitive indexes (without proving statistical tests or with statistical arguments) were selected. The studies included in this review are classified into two main study types: (1) Investigating the effects of using virtual reality-based systems by performing statistical tests, and (2) Investigating the effects of using virtual reality-based systems without complex statistical calculations (measuring the effectiveness by calculating central or dispersion indexes such as mean and standard deviation). Therefore, due to the heterogeneity of the studies in terms of methodology, statistical analyses and outcomes, meta-analysis was not possible in this study, so a narrative synthesis was used to describe and compare the paper's results. To conduct synthesis, the included papers were categorized based on various characteristics, such as bibliographic information, sample size and description, experimental interventions, study design, cognitive outcomes, assessment times, scores, and effectiveness of applied systems. Similar to a systematic review conducted by Farzandipour et al. [19], the effect of virtual reality-based interventions was classified as being significantly positive, positive without statistical argument, and having no effect (not statistically significant).

### Quality assessment of the selected studies

The quality of screened papers was assessed by the Effective Public Health Practice Project (EPHPP) quality assessment tool [20, 21]. The EPHPP is a proper tool for evaluating diverse study designs such as Randomized Clinical Trials (RCTs), Non-Randomized Clinical Trials (Non-RCTs), Observational Studies With Controls (OWCs), and observational studies without controls [22]. The EPHPP includes domains for assessing internal and external evidence validity in studies or model validity assessment in RCTs or Non-RCTs. This tool comprises six sections, including selection bias, study design, confounding variables, blinding, data collection methods, and withdrawals and dropout. Each criterion is graded as strong, moderate, or weak, and then the overall quality score (global ratings) is measured for each study. Studies with two or more weak ratings are given a global rating of weak, studies with one weak rating are given a global rating of moderate, and studies with no weak rating are given a global rating of strong. Two researchers (SR and LS) independently scored each study, and disagreements were resolved through discussions among the researchers.

## Research results

### Results of literature search

A total of 954 papers were extracted from the primary searches in scientific databases, and after removal of duplicates, 688 papers remained for further assessment. Finally, only 17 articles that met the inclusion criteria were entered the review.

### Characteristics of the selected studies

**Fig. 3:**
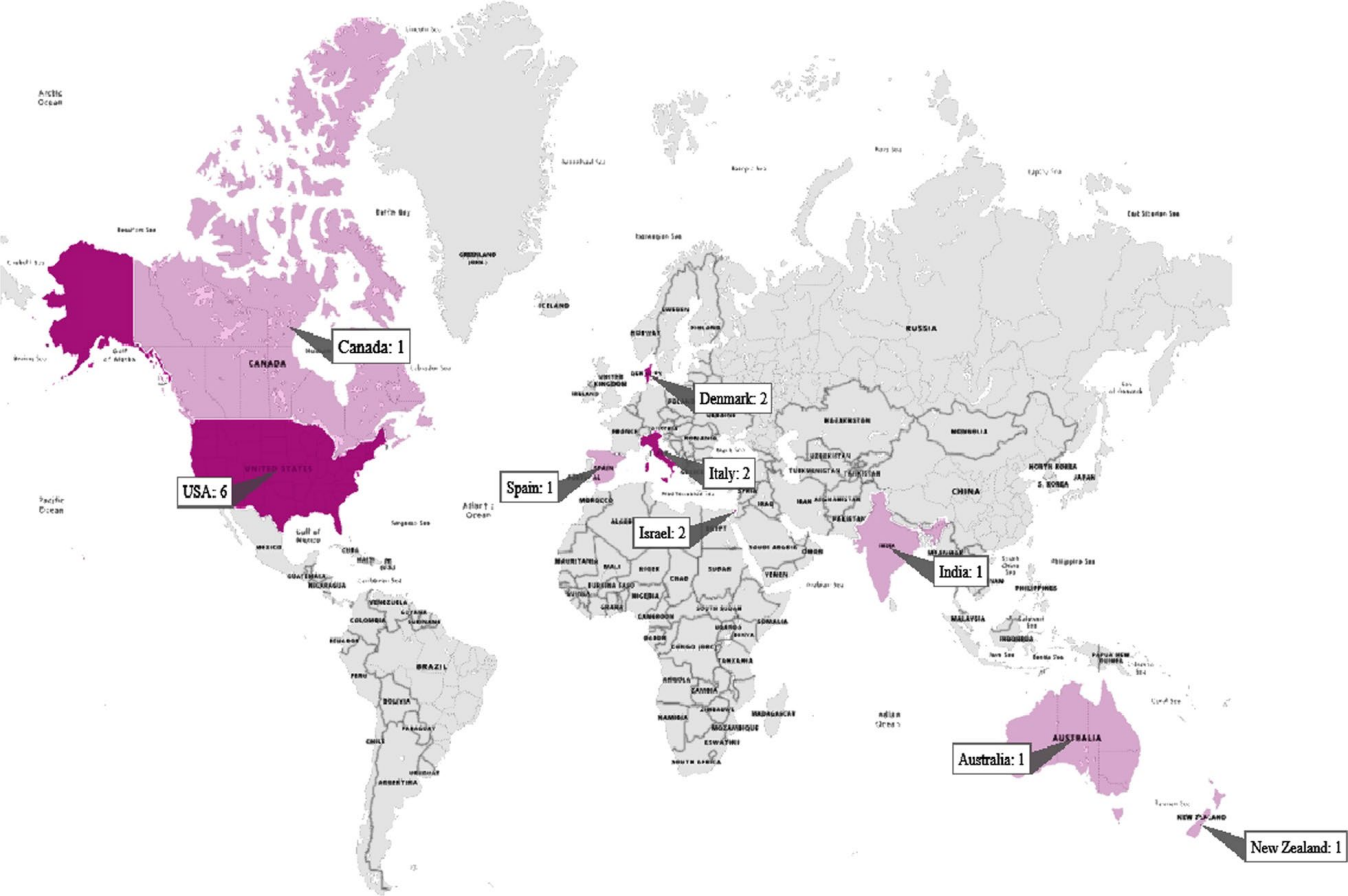
The distribution of articles based on country

The key characteristics of selected studies are summarized in Table 2. Most of the selected studies 35% (6/17) had been conducted in the USA. The distribution of papers based on countries is presented in Fig. 3. Screened papers had been published between 2007 and 2021. Five studies had been conducted in 2019. A total of 226 autistic patients had participated in all 17 studies. The sample size in the selected studies ranged from 1 to 56 participants (Median: 8, Q1: 3.5, Q3: 15.5). The majority of participants in the selected papers were male (85.05%), and their mean age ranged from 6 to 44 years. The number of intervention sessions ranged from 1 to 24 sessions, with the time of each session being varied (minutes). A description of experimental interventions for each article is reported in Table 2. Meanwhile, four studies were observational with a control group (case–control), ten were observational without a control group (pre-post interventions), and three were RCTs.

**Table 2:** The characteristics of reviewed articles (n = 17)

Authors	Sample size	Sample description (Sex- age)	Experimental intervention	Virtual reality implementation	Study design	Session details (number of sessions, duration)	Cognitive outcomes (memory, attention, executive function (daily skills), learning)	Assessment time	Assessment Score	Results	Reported limitations
De Luca et al. 2019, Italy [23]	1	16-year-old boy	VR training using an innovative tool, namely BTS-Nirvana (BTS-N)	BTS-N is a medical device based on VR, this is the first device using a two-dimensional flat-screen projection system with optoelectronic infrared sensors, through which the patient can simply interact by his movements	Pre and post test	24 session, each session 40 min	Attention and visual-spatial exploration	Baseline and post intervention	Authors found a significant increase of attention processes, by the improvement of MTCM [from T0 with a rapidity score of 5 c¼49, 2–3 SD and accuracy score of 34 c¼124, 7 > 3 ds to T3 with a rapidity score of 19 c¼49 and accuracy score of 68 c¼124,7 > 3 ds]	This case-study showed that VR could be helpful to potentiate cognitive and adaptive behavioral (with regard to attention process, spatial cognition, and visual-motor integration	Epidemiological bias Impossibility of causal inference Generalization, and over-interpretation Small sample sizes Cost of virtual reality devices
Wang et al. 2013, Canada [24]	4	AgeM = 6.7 yrs., Male = 3, Female = 1	Virtual reality training programs	This program is based on a two-dimensional flat screen projection system. This system has motion-capture capabilities, where a tracking camera is able to capture and project a child’s image and motions on screen in real-time	Pre and post test	4–6 sessions	Contextual processing of objects	Baseline, middle of intervention and a two-week follow up session	The results demonstrate improvements in contextual processing ability from baseline to treatment for each child, with average increases from 15% (Child 2) to 46% (Child 4). All children maintained a high level of performance at the two-week follow-up assessment	All children demonstrated statistically significant improvements in contextual processing and cognitive flexibility. Mixed results were found on the control test and changes in context-related behaviors	Small sample sizes The lack of multiple, independent assessors Time of follow up duration is limit Potential eye damage
Lamash et al. 2017 Israel [25]	56	AgeM = 14.58 yrs., Male = 29, Female = 4	VAP-S	V AP-S—is a virtual environment software that operates on a laptop or desktop and requires the use of keyboard arrows and a mouse	Case–control study	8-sessions	Executive function: teach shopping task form a supermarket	Baseline and post intervention	The results show that among the intervention group, significant improvements were found in the attention component (P < 0.01) and in the executive functions component (P < 0.01); the intervention group showed a significant improvement in all the accuracy indices in shopping task (F (1, S4) = 14.23 P < 0.01)	The results show improvement of the intervention group compared to the control group in several indices, indicating great promise for intervention programs based on virtual technology for improving the independency and community participation with ASD	No limitation was reported
Adjorlu et al. 2019, Denmark [26]	5	18 to 22 yrs., Male = 5	VR for teaching money skills	The VR money training intervention was developed using Unity 3D and C# scripting. The application was developed to run on the HTC Vive VR hardware The virtual coins and bills were designed using textures from images of real danish money; The coins and bills could be grabbed by the player using the grab button on the HTC Vive controller and released agains by releasing the same button	Pre and post test	5 sessions for 10–15 min each one	Executive function: teach money skills	Baseline and post intervention	Results showed that the maximum improvement was score from 9 pre-test to 30 post-test and minimum score was 0 to 0. Students C and D illustrated very small improvements (from 3 to 8 and 0 to 2 correct purchases)	Four out of the five participants showed some improvement in their money skills after five training sessions with the VR application	No limitation was reported
Adjorlu et al. 2017, Denmark [27]	9	12 to 15 yrs., Male = 8, Female = 1	VR based supermarket shopping training system	The VR intervention was developed using Autodesk Maya and Unity. HTC Vive was chosen to run the application due to its effective room scale tracking. The signals are than captured via the infrared sensors placed on the VIVE head-mounted display	Case–control study	7-sessions	Executive function: teach shopping task form a supermarket	Baseline and post intervention	The treatment group efficiency score was 100% during both the baseline- and post-treatment assessments. On the contrary, the control group effectiveness decreased from 97% in the baseline assessment to 91% in the final assessment	The study indicates some positive effects of a head-mounted display-based VR simulation to train DLS of individuals	The weight of devices
Bian D et al. 2019, USA [28]	23	AgeM = 15.19 yrs., Male = 21, Female = 2	VR driving simulator	Models in the virtual driving environment, such as traffic lights, stop signs, and vehicles, were developed with the modeling tools ESRI CityEngine and Autodesk Maya. The game development platform Unity3D was used to implement the system logic	RCT	1-session, 90 min	Executive Function: teach driving task	Baseline and post intervention	No differences were found in performance data, however, with participants in ES group achieving similar performance as participants in PS group	These findings could support future work into driving simulator technologies, which could provide opportunities to practice driving skills in cost-effective, supportive, and safe environments	Long-term driving training program is needed
Cox et al. 2017, USA [29]	51	AgeM = 17.96 yrs	VRDST	The commercially available DGS-78 VRDS is a realistic driver’s cockpit with side and rear-view mirrors. The driver’s view is projected onto a 2.44 m (8 ft) diameter, 210° curved screen	RCT	8–12 sessions, 60 min each session	Executive Function: teach driving task	Baseline and post-intervention and after 3 months of training (follow up duration)	The general tactical composite score improved differentially across groups (p < 0.010), and a significant covariate (p < 0.001, β = 0.50) indicated that better baseline performance was associated with better post-assessment performance	VRDST significantly improved driving and EF performance over RT. This study demonstrated feasibility and potential efficacy of VRDST for novice ASD drivers	Obtrusive or irritating nature of wearing eye-tracking glasses Time of follow up duration is limit
Peisley et al. 2019,New Zealand [30]	4	6 to 7 yrs	Virtual Week	Participants viewed the Virtual Week board game on a laptop PC monitor and responded using the keyboard. Participants clicked the mouse to roll the die, read aloud the event cards, and made decisions about the daily activities. All children were reading at, or slightly below, their age level and could read the cards without assistance	Pre and post test	45–60 min each session	Memory	Baseline and post intervention	There was a significant main effect of regularity on mean accuracy scores and prospective memory, p = .04, r = .79, but no main effect of PM-task type, p = .59, r = .27, and no significant interaction between regularity and task type, p = .08, r = .65	These results suggest the delivery of positive reinforcement may improve accuracy on PM tasks post reinforcement	Long-term training program is needed
Austin DW et al. 2008, Australia [31]	2	AgeM = 14.50 yrs., Male = 29	VRH	The VRH method uses a head-mounted display to create a non-threatening, virtual reality environment, where the hypnotherapeutic process can be implemented	Pre and post test	4-sessions	Attention	Not mentioned	No Scores are reported	They indicated that they believed it was an effective technique to gain their son’s attention, and this, combined with the fact that the boys found it enjoyable and engaging, led them to believe there is significantly potential for this particular treatment modality	No limitation was reported
Herrera et al. 2008, Spain [32]	2	AgeM = 15.8 yrs	VE	Not mentioned	Pre and post test	Not mentioned	Executive Functions	Baseline and post intervention	The first participant showed considerable progress in structured pretend play, obtaining an improvement of 6.5. The advances shown by the second participant were also in both types of play. In the structured play test, he gained 4.75 points (from 40.3 to 49.8 months)	The results, confirmed by independent observers, showed a significant advance in pretend play abilities after the intervention period	No limitation was reported
Josman et al. 2008, Israel [33]	12	8 to 12 and 14 to 16 yrs., Male = 10, Female = 2	VE	Three keyboard keys (marked on a standard keyboard with round, colored stickers) were used to change the user's viewpoint to the right or to the left or to initiate street crossing. Users who succeeded in safely crossing the street automatically proceeded to the next stage	Case–control study	Not mentioned	Executive Function: teach street-crossing skills	Baseline and post intervention	The maximum stage reached by the research group during Phase A ranged from one to four, with a mean of 2.7 (SD) = 1.2). Using the Wilcoxon test, p < .01 was obtained, demonstrating a significant difference between the two groups	Significant differences were found between the performance of the experimental and control groups within the VE. half of the experimental subjects made considerable improvement	No limitation was reported
Weilun et al. 2011, Singapore [34]	9	AgeM = 16 yrs	Virtual game	Following an iterative and incremental model for software development life cycle, an interactive quiz programme which employs virtual avatar to pose academic-related questions to autistic students is developed. Additionally, drawing on a Sumo wrestling game using robotics agent iRobot Create to hone motor skill is evaluated	Pre and post test	Not mentioned	Learning meaningful academic activities	Not mentioned	No scores were reported	Experimental results showed that the deployment of virtual games hold great potential in motivating and exciting the children in their learning process, as well as providing valuable insights to related rehabilitative industries	No limitation was reported
Self et al. 2007, USA [35]	8	6 to 12 yrs., Male = 6, Female = 2	VE	Not mentioned	RCT	20-sessions, 30 min each session	Executive Function: teach how to escape the fire and survive	Baseline, middle of intervention and post intervention	No scores were reported	Both groups improved in their learning and transfer of safety skills. The VR group, however, learned these skills in considerably less time	No limitation was reported
Saiano et al. 2015, Italy [36]	6	19 to 44 yrs., Male = 7	VE	The experimental apparatus included a video projector, displaying a virtual reality environment on a 2 m × 2 m screen. The participants were required to stand in front of the screen, at a distance of approximately 2 m. Instead of using mouse and keyboard to interact with the VE, they used a markerless motion capture device (Microsoft Kinect), placed below the screen to record the subjects’ full-body movements in 3D space	Pre and post test	10-sessions, 45 min each session	Executive Function: teach street-crossing skills	Baseline and post intervention	The ability to follow the street signs, we found that subjects significantly increased (p = 0.0042; paired-samples t-test) their average speed from T0 to T1, of an amount ranging from 40 to 100%; they found no significant changes in path length, figural distance, and composition index	The six subjects who completed the protocol easily learned the simple body gestures required to interact with the VE. Both parents and caregivers reported a significant improvement in the subjects’ street crossing performance	Small sample sizes The team only made an indirect assessment of transfer of the learned skills to real life
Dixon et al. 2019, USA [37]	3	AgeM = 6.6 yrs., Male = 3	VE	Not mentioned	Pre and post test	3 and 5 min, an average of 5.46 trials; up to 2 to 3 days in a 1-week period	Executive Function: teach street-crossing skills	Baseline and post intervention	For each participant in the study, low, stable scores were observed during baseline and an increase in scores was seen after each VR training condition	Findings suggest that immersive VR is a promising medium for the delivery of safety skills training to individuals with ASD	Lack of testing in the natural environment Only the skill of identifying whether a street is safe to cross was trained and evaluated Supervised usage and limited access to such technology until larger studies demonstrate its safety
Wade et al. 2017, The USA [38]	Pilot 1: 7 Pilot 2: 9	Pilot 1: AgeM = 16 yrs., Male = 4, Female = 3 Pilot 2: AgeM = 15.26 yrs., Male = 9	VADIA	Users interact with the system via a Logitech G27 controller, which features a steering wheel, pedal board, and gear shifter, although this last item was not utilized in the presented studies; The G27 controller mounts conveniently onto a car-like bucket seat that is positioned in front of a flat panel LCD monitor displaying the driving environment	Case–control study	Pilot1: 90 min, 1 session Pilot 2: 60 min, 6 session	Attention	Baseline and post intervention	Study 1 demonstrates statistically significant performance differences between individuals with (N = 7) and without ASD (N = 7) with regards to the number of turning-related driving errors (p < 0.01) Study 2 shows that both the performance-based feedback group (N = 9) and combined performance- and gaze-sensitive feedback group (N = 8) achieved statistically significant reductions in driving errors following training (p < 0.05)	In this study, the use of virtual reality-based systems has significant effects on people's cognition index but researcher team will assess changes in performance based on best practice clinical and on-road evaluation metrics	Small sample sizes Limit the statistical power of the analyses and subsequently the generalizability of the results Age and driving experience are not sufficiently controlled (confounders are not completely controlled) Potential addiction
Bozgeyikli et al. 2016, USA [39]	15	AgeM = 25.4 yrs	VR4VR	The system is composed of the following hardware components: a Head Mounted Display (HMD); an optical motion tracking system with 12 cameras; a large 180° curved curtain screen; controllers; tangible objects equipped with optical markers that can be tracked real time by the system; and a tablet computer for remote control panel for the job coaches	Pre and post test	Not mentioned	Executive function: teach money skills, cleaning, vocational training, shelving	Not mentioned	No scores were reported	Proposed system utilizes six transferrable skill modules within immersive virtual environments for vocational training of individuals with ASD	No limitation was reported

MTCM: The Modified Little Bell Test; SD: Standard Deviation; VAP-S: The Virtual Action Planning Supermarket; VR: Virtual Reality; DLS: Daily Living Skills; RCT: Randomized Control Trial; PS: Performance-Sensitive System; ES: Engagement-Sensitive System; VRDST: VR Driving Simulation Training; PC: Personal Computer; DGS-78: Driver Guidance System; PM: Prospective Memory; VRH: Virtual Reality Hypnosis; VE: Virtual Environment; VADIA: The Virtual Reality Adaptive Driving Intervention Architecture; VR4VR: Virtual Reality system For Vocational Rehabilitation; VADIA: The Virtual Reality Adaptive Driving Intervention Architecture

### Quality assessment of the included papers

**Fig. 4:**
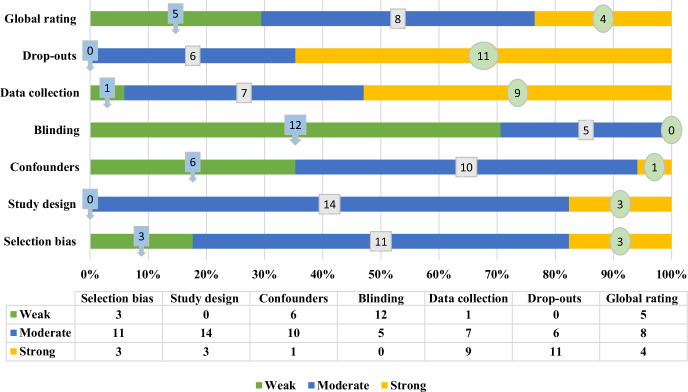
Quality assessment of the selected papers

The quality of screened studies is presented in Fig. 4. Based on the analysis, most studies were strong in terms of drop-outs (64.70%) and data collection (52.94%), moderate in terms of study selection (82.35%), and confounding variables (58.82%), and weak in terms of blinding (70.58%). According to global rating scores, 29.41% of the studies were weak, 47.05% were moderate, and 23.52% were strong in terms of quality. Details of quality assessment are presented in Appendix 1, Table 4.

### Experimental interventions

The virtual reality training programs and environments were the main interventions in the selected studies. In two studies (a case–control study and a pre-test/post-test study) virtual reality-based systems were used to teach financial, cleaning, vocational, and shelving skills. Patients in these studies received five sessions of virtual reality-based training that took about 10–15 min each [26, 39]. Moreover, in two case–control studies, supermarket shopping training systems were developed through virtual reality environment. In these studies, executive functioning like teaching how to conduct shopping was taught, and in other study, patients received seven and eight sessions of cognitive rehabilitation [25, 27]. Additionally, in one case–control and two pre-test/post-test studies, virtual environments (VEs) were designed for teaching street-crossing skills. In some studies, patients received five or ten rehabilitation sessions [36, 37]. In two RCTs, virtual reality driving simulators were developed. In these trials, the targeted cognitive outcome was rehabilitating executive functioning like improving driving skills in real and immersive environments [28, 29]. In another RCT, how to escape and survive a fire were trained in virtual environment, so that participants received 20 sessions of training that took about 30 min each [35]. Furthermore, in one case–control and two pre-test/post-test study, attention and visual-spatial exploration were rehabilitated by virtual training programs, and the number of training sessions received by patients was different [23, 31, 38]. In four pre-test/post-test studies, contextual processing of objects, memory, various executive functions and meaningful academic activities were rehabilitated and improved, respectively [24, 30, 32, 34].

### Effects of interventions on outcomes

The results of each paper are presented in Table 3. Each study targeted different cognitive indexes. Cognitive rehabilitation refers to a wide range of evidence-based interventions designed to improve cognitive functioning, restore normal functioning and compensate for cognitive deficits in brain-damaged or cognitively impaired individuals. Therefore, cognitive rehabilitation is used to improve individuals' psychological, social, mental, and functional cognition. In our selected studies, cognitive indexes such as attention, executive functioning, and memory had been improved. Eleven studies had examined executive functioning or daily skills by using various assessment tools. In three studies, attention had been assessed at baseline and post-intervention time, and in three studies, memory, academic activities, and contextual processing of objects had been improved and assessed by different assessment tools. Table 3 presents a summary of used systems’ effectiveness (1. positive and statistically significant, 2. positive without statistical argument, 3. no effect (not statistically significant). The frequency distribution of studies by cognitive outcomes is also provided. As seen in Table 3, nine studies reported statistically significant improvement in cognitive outcomes, and seven studies did not report any statistical test for improvement assessment, but they provided positive arguments along with measuring the effectiveness by calculating dispersion or central indicators. In only one study, there were no statistical or non-statistical improvements in cognitive indexes.

**Table 3:** A summary of the employed systems’ effectiveness by cognitive outcomes

Effectiveness: (1. statistically significantly positive, 2. positive without statistical argument, 3.not effective (not statistically significant))	Column Labels			
Row Labels	No effect (Not statistically significant)	Positive without statistical argument	Statistically significant positive	Grand Total
Attention		1	1	2
Attention and visual-spatial exploration			1	1
Contextual processing of objects			1	1
Executive function: teach driving task	1		1	2
Executive function: teach how to escape the fire and survive		1		1
Executive function: teach money skills		1		1
Executive function: teach money skills, cleaning, vocational training, shelving		1		1
Executive function: teach shopping task form a supermarket			2	2
Executive function: teach street-crossing skills		1	2	3
Executive functions		1		1
Learning meaningful academic activities		1		1
Memory			1	1
Total	1	7	9	17

## Research discussion

The main objective of this review was to analyze and identify the studies conducted on the use of virtual technologies in the cognitive rehabilitation of autistic children and adults. To do this, we selected 17 studies based on our inclusion and exclusion criteria. The article's principal aim was to examine the virtual reality-based technologies that can improve cognitive indexes such as executive functioning, attention, memory, learning, and daily skills. In this regard, it should be mentioned that the most popular immersion technology was virtual reality which had relatively positive effects on the cognitive outcomes of autistic persons. The results showed that in most of the selected studies, males were more likely to participate in the study. According to Table 2, the majority of participants in reviewed citations were male (85.05%). Various studies, along with anecdotal evidence, suggest that the ratio of autistic men to women ranges from 2:1 to 16:1; therefore, ASD is more than four times more common among males than females. In this regard, the most up-to-date estimate is 3:1 [40].

The reason for this phenomenon is unknown, but it is logical to conclude that it has something to do with the male–female gender differences. Some others believe that autism and attention disorder affect girls differently than boys, as girls may show less restricted interests, repetitive behaviors, and cognitive defects than boys [41]. The estimations made all over the world indicate that the prevalence of ASD in boys is higher than in girls. Also, according to our research, 35% of the studies used in this review were conducted in the United States, which could indicate a high prevalence of autism in this country. In 2020, it was estimated that around 222 per 10,000 children in the United States had autism spectrum disorder, one of the highest prevalence rates in the world [42].

Following the increase in the number of children and adolescents with ASD, the United States tried to design the most advanced technologies to solve this problem. The American Autism Association tries to make as many opportunities as possible for individuals and families affected by autism. Given that the United States is a developed country, it is expected that it would design innovative technologies such as virtual reality for people with autism [40]. These technologies, which have been designed and used in various studies, have shown that they can significantly affect the cognitive function of patients*.* Thus, the combination of immersive virtual technologies and cognitive problems has led researchers in countries such as the United States to develop virtual reality-based systems for the cognitive rehabilitation of people with autism.

Based on our results, 16 studies had shown positive effects of virtual reality-based systems on the cognitive functions of autistic people. Among these studies, nine showed a significant and statistical effect of interventions on the cognitive indexes of people with autism. Other studies showed that systems that do not require the use of heavy tools to annoy autistic patients could have a positive effect on their cognition [23, 30–32]. The use of comfortable devices (such as glasses or physiological sensors) in designed technologies encourages autistic patients to use and easily tolerate them during treatment sessions. One of the most critical limitations reported by the selected studies was the intolerance of virtual reality glasses by children with autism. However, choosing children with the right age range and higher Intelligence Quotient (IQ) was one of the solutions used to deal with this limitation. In addition, the cost of designing and manufacturing immersive technologies was one of the most important issues for researchers to be able to build the system in the best possible way. Providing such expensive systems for all mental health centers is also impossible.

The most critical limitations reported by the researchers included the small sample size, the need to design randomized clinical trials or interventional studies, the need to use long-term training program, and short follow-up time [33, 43–45]. In addition, to accurately identify the impact of designed systems, they must be used in a therapeutic environment. Accurate identification of cognitive problems in people with ASD is an important step that must be taken before designing and applying emerging technologies such as virtual reality in the real environment. According to the recommendations presented in the selected papers, needs assessment and identification of system requirements in the pre-development and implementation stages are considered key factors [17, 46, 47].

According to the results of the evaluation obtained from the "Effective Public Health Project (EP HPP)" checklist, the blinding approach was weak in most studies (12 studies) and moderate in five studies. Participants (in different age ranges) in the selected studies had been subjected to cognitive rehabilitation by virtual reality technology. These individuals were generally aware of the type of intervention, and it was impossible to blind the participant from the research question. Also, since some participants were children, their parents had to provide informed consent. On the other hand, the rehabilitation team was aware of the type of intervention to guide the participants on how to use the technology, so blinding them was impossible. Only in a limited number of studies, the outcome assessor or analyst was unaware of the research question or intervention status, but in others, the outcome assessor(s) was aware of participants' intervention status. In 11 of the reviewed studies, drop-outs reporting was rated strong, and six citations rated it as moderate. In these studies, withdrawals and drop-outs mostly reported in terms of numbers and/or reasons per group, and the percentage of participants completing the study were indicated. Also, in nine studies, the data collection method was rated strong, and in seven, it was rated moderate. In most studies, the data collection tools were shown to be valid and reliable.

### Limitations and strengths of this study

This study had several strengths. One of the strengths of this study was its search that was carried out in valid databases, including Medline (through PubMed), Scopus, ISI Web of Science, and IEEE Xplore. This comprehensive scientific search enabled us to cover almost all papers published in this field. Meanwhile, we also did not impose any time limit on the search strategy. Two authors independently extracted data and assessed the quality of studies. A valid and comprehensive tool was used to assess the quality of selected studies.

We have also encountered some limitations in this study. The difficulty of comparing studies due to the heterogeneity of the results, and the exclusion of published studies other than English language ones were among the limitations and challenges of this study.

## Conclusion

This systematic review revealed the importance of using different virtual reality-based approaches to improve the cognitive indexes of people with ASD. By applying a systematic approach, the authors provided an exhaustive overview of the use of virtual technologies that could rehabilitate cognitive indexes such as executive functioning, attention, memory, and daily skills. This survey showed that virtual reality-based approaches have the potential to improve the cognitive indexes of people with ASD. Meanwhile, the results of this study can encourage researchers to use the new immersive approaches to rehabilitate defects in autistic people. However, further studies are needed to investigate the real effects of these technologies and their effectiveness in the long run.

## Data Availability

All data generated or analyzed during this study are included in this published article.

## Appendix

See Table 4.

**Table 4:** Quality of the included studies

Study	Selection bias	Study design	Confounders	Blinding	Data collection	Drop-outs	Global rating
[26]	Weak	Moderate	Weak	Weak	Strong	Strong	Weak
[27]	Moderate	Moderate	Moderate	Weak	Strong	Strong	Moderate
[28]	Strong	Strong	Moderate	Weak	Strong	Strong	Moderate
[29]	Strong	Strong	Strong	Moderate	Strong	Strong	Strong
[30]	Moderate	Moderate	Moderate	Weak	Moderate	Strong	Moderate
[36]	Strong	Strong	Moderate	Moderate	Strong	Strong	Strong
[31]	Moderate	Moderate	Moderate	Weak	Strong	Strong	Strong
[33]	Moderate	Moderate	Moderate	Weak	Moderate	Moderate	Moderate
[34]	Moderate	Moderate	Weak	Weak	Weak	Moderate	Weak
[35]	Moderate	Moderate	Weak	Weak	Moderate	Moderate	Weak
[37]	Moderate	Moderate	Moderate	Weak	Strong	Strong	Moderate
[23]	Weak	Moderate	Moderate	Weak	Strong	Moderate	Weak
[24]	Moderate	Moderate	Weak	Moderate	Moderate	Moderate	Moderate
[25]	Moderate	Moderate	Moderate	Weak	Moderate	Strong	Moderate
[32]	Weak	Moderate	Weak	Moderate	Moderate	Moderate	Moderate
[38]	Moderate	Moderate	Moderate	Moderate	Strong	Strong	Strong
[39]	Moderate	Moderate	Weak	Weak	Moderate	Strong	Weak
